# Chiropractic student choices in relation to indications, non-indications and contra-indications of continued care

**DOI:** 10.1186/s12998-017-0170-y

**Published:** 2018-01-23

**Authors:** Stanley I. Innes, Charlotte Leboeuf-Yde, Bruce F. Walker

**Affiliations:** 1 School of Health Professions, Murdoch University, Murdoch, Australia; 2 Institut Franco-Européen de Chiropraxie, Ivry sur Seine, France; 3 CIAMS, Université Paris-Sud, Université Paris-Saclay, 91405 Orsay Cedex, France; 4 CIAMS, Université d’Orléans, 45067 Orléans, France; 5 Institute for Regional Health Research, University of Southern Denmark, DK-5000 Odense C, Denmark

**Keywords:** Clinical decisions, Diagnosis, Chiropractic, Education

## Abstract

**Background:**

The quality of health care provider clinical decisions has long been recognized as variable. Research has focused on clinical decision making with the aim of improving patient outcomes. No studies have looked at chiropractic students´ abilities in this regard.

**Method:**

In 2016, advanced students from two Australian chiropractic programs (*N* = 444) answered a questionnaire on patient case scenarios for neck and low back pain (LBP). We selected 7 scenarios representing the three categories; continuing care, non-indicated care, and contraindicated care. This represented a total of 21 tested scores. Comparisons of correct answers were made a) for program years 3, 4 and 5, and b) between the three categories of care.

**Results:**

In almost 1/3 of scenarios, correct scores were 70% or greater. Best results were for two neck pain cases (simple and with spinal cord involvement). Continued care showed most improvements with study year. However, the scenarios that reflected non-indication for continued care had much worse results and did not improve in higher years. For the obvious contraindicated neck scenario, the results were good from the beginning and progressively improved and for a contraindicated LBP scenario the results started poorly in year 3 but improved over the program years.

**Conclusions:**

Although student responses were generally good, there is still room for improvement, especially for non-indicated care. The quality of students’ clinical decisions can be measured and thus has the potential to be used by chiropractic educators and regulatory bodies to identify student’s in need of assistance as well as to monitor chiropractic programs in relation to student competence.

**Trial registration:**

Not applicable.

**Electronic supplementary material:**

The online version of this article (10.1186/s12998-017-0170-y) contains supplementary material, which is available to authorized users.

## Background

Quality care is a key aim for all healthcare systems [1, 2]. The most common domains used to measure healthcare performance are safety, effectiveness and access [3]. Undergraduate education for healthcare providers aims to produce competent graduates who can implement evidence-based and common sense care in order to meet expectations associated with safety and effectiveness [4].

Chiropractic students in Australia undergo programs which are 5 years in duration [5]. Like medical students, the early years are spent learning the basic sciences, after which they progress to the clinical sciences with the expectation that they will learn to apply this knowledge and make reasonable clinical decisions.

Clinician judgment has been described as being notoriously fallible, irrational and difficult to comprehend [6, 7]. As a result medical research has increasingly emphasized the need for evidence based medicine [8]. This is similar for many first or primary contact practitioners [9]. The difficulty of incorporating evidence continues to be a struggle and education is viewed as an important part of the solution [10]. To this end medical education authorities commonly use milestones for the purpose of standardizing expectations and providing clarity for both assessors and learners about their development across competency domains [11]. Information about this progression could be used to facilitate decisions regarding the educational quality and effectiveness of chiropractic programs. Further, it may identify directions for remedial intervention to assist chiropractic students, who are not making satisfactory progress.

While there are some variations between chiropractic regulatory authorities’ accreditation standards, one common standard is the expectation that chiropractic undergraduate institutions will produce graduates capable of making decisions which are in the best interest of their patients [12–14]. They should for example be able to determine when treatment is indicated and when not. Thus they should be able to distinguish between indications, non-indications and contra-indications. Conditions indicated for treatments should be plausible, often experienced as successfully treated, and when possible evidence-based. Non-indications are conditions that are unlikely to respond in a positive manner to chiropractic treatment whereas contra-indications are those that might worsen with such treatment [15]. Thus back pain caused by an episode of dysmenorrhea is a non-indication, as it is not amenable through usual chiropractic care, whereas back pain caused by a spinal metastatic lesion is a contra-indication, as it could worsen (bony fracture) with a manipulative thrust.

During the course of treatment, chiropractors must also be able to make common sense choices of when they should continue and when they should stop. Conditions that do not improve, or progress to develop warning signs of contra-indications, should not continue to be treated. Non-indicated treatment, even if not contra-indicated, brings with it an unnecessary cost and reflects badly on any professional or profession. On the other hand, continued care, also called ‘maintenance care’ in the chiropractic context, is a logical choice in cases with a previous recurring history and good response to treatment [16, 17].

A word search by the authors of the five Councils on Chiropractic Education (CCEs), who are the regulatory agencies of the education and competency standards for chiropractic programs, found occurrences of the words ‘indications’ and ‘contraindications’ but none for ‘non-indications’. The authors contend that this aspect of training requires a detailed discussion of all three terms. Further, that this is overdue and it would be relevant to start exploring how or if these issues manifest in the current educational setting.

It is reasonable to expect that chiropractic students, at an appropriate stage of training, attain the same level of good / correct clinical decision making as graduated chiropractors and that this would be a graded and positive process. Also likely is that students within each year may not uniformly attain this ability. For example, contraindications to stop care may be learned early on, but the recognition to stop non-indicated care may occur in later years with clinical experience.

Accordingly, our research questions were;

1. Is there a progression in chiropractic students abilities in different years of the program to correctly identify

- The continuation of ‘indicated’ care in relation to a neck pain case with two scenarios and a LBP case scenario?
- The cessation of ‘non-indicated’ care in relation to a low back pain case with two scenarios?
- The cessation of ‘contraindicated’ care in relation to a neck pain case scenario and a LBP case scenario?

2. Is students’ knowledge in each year of the program the same or different for understanding when treatment is ‘indicated’, ‘non-indicated’ and ‘contraindicated’.

- In year 3
- In Year 4
- In Year 5

## Methods

### Procedure

This is a secondary analysis of a study that has been reported in full elsewhere and is available as free full text online [18]. Two questionnaires on neck pain [19] and low back pain [20] were included in a survey on the association between psychological profiles and practice patterns, conducted towards the conclusion of the academic year (October and November) in 2016 [18]. The questionnaires have been previously used to assess chiropractors’ clinical decision-making profiles [19–22]. Participants were chiropractic students from two chiropractic programs at two universities (Programs A and B) in Australia and the original research project required data from all the years [18]. For the purposes of this present study, only data from the third to the fifth years were included, as it was deemed that students in Years 1 and 2 did not have the clinical knowledge to understand the case scenarios used.

Ethics approval was granted by Murdoch University (Project No 2016/118) and the project was classed as a negligible risk research. The project followed the same protocols in both institutions, consent was obtained from students, data were non-identifiable (anonymous) and permission was obtained from the Head of the other chiropractic program to conduct the survey. Accordingly, the study met the criteria for classification under the Australian National Statement on Ethical Conduct of Human Research (2007) (Sections 5.1.8 and 5.1.22) as exempt from requiring ethics approval from both universities.

### Information used for this study

Information on various demographic details of the participants was obtained (chiropractic program, sex, year of study).

### Survey instruments

#### Neck pain survey

In the first case study [19], five neck pain scenarios were presented, beginning with a simple uncomplicated case of neck pain and progressing through to a scenario requiring immediate medical referral (Additional file 1). The case consisted of the following general information: “A 28-year old man, tennis player by profession, consults you for a right-sided intense neck pain without any radiating pain. You note an antalgic position of the head, no other musculoskeletal signs (no torticollis), no other health problems in particular, normal x-rays for his age, and no signs of serious pathology (red flags)”. There was a choice of six answers for each of the five scenarios ranging from the chiropractor treating the patient on their own, through to not providing treatment and arranging referral.

This questionnaire was originally designed by three 4th year chiropractic students, a lecturer in clinical sciences, and three lecturers in research methodology in France. French chiropractic academics proof-read the document for logic and absence of errors. Low percentages of “no response” in the original study indicated it was easy to understand and respond to. The questionnaire was translated into plain English for the purposes of publication and this version was used in our present survey.

The progression of this case was straightforward and the distinction between the simplest to the most severe case was clear, making it easy to define suitable and non-suitable clinical choices. Consensus was demonstrated in the previous study on the most appropriate management or ‘indicated’ choice across the five scenarios [19].

We selected scenarios 1 and 2 for the purposes of this study. Here the patient presented with simple uncomplicated neck pain. The continuation of only chiropractic care was clearly indicated. Consequently, it was designated as the ‘indicated’ or ‘correct’ choice.

We also selected scenario 5 for ‘contraindicated’ purposes. In this scenario the patient had been resistant to treatment and there was clear evidence of progressive neurological deterioration and symptomatology. Selection of any option other than the referral choice was deemed to be ‘contraindicated’ (the full rationale is seen in Additional file 1).

#### Low back pain survey

The second case study described a range of clinical scenarios for a patient with low back pain (LBP) and designed to find out which management strategies chiropractors would prefer to use [20] (Additional file 1). This questionnaire included nine possible outcomes that were briefly described. These nine clinical scenarios differed both on past history and reaction to treatment. An identical set of six clinical management alternatives were offered for each of the nine outcome scenarios, of which the respondents should choose one alternative for each scenario.

The LBP questionnaire was previously designed, written, distributed, answered and subsequently adjusted in English by a research team consisting of 7 chiropractors, with clinical experience ranging from 4 to 25 years, who obtained their chiropractic degrees in English speaking countries. They were supervised by a chiropractic researcher. The term ‘treatment’ used in the questionnaire was purposefully not defined so that it aligned with previous studies used [23].

In the first LBP survey conducted on Swedish chiropractors, a pattern of self-reported clinical management strategies was demonstrated which allowed identification of those who did and did not follow ‘clinically logical’ answers for this hypothetical case (Additional file 1) [20]. This was followed by a smaller interview study in Denmark using the same questionnaire which revealed the same pattern [21]. Thereafter, the same survey was conducted on French chiropractors, again, revealing a similar pattern [22]. The Swedish and Danish chiropractors responded to the questionnaire in English and the French chiropractors did so in French after a double translation (English to French; French to English).

The basic facts for this hypothetical patient were: “A 40-year old man consults you for low back pain with no additional spinal or musculoskeletal problems and with no other health problems. His X-rays are normal for his age. There are no ‘red flags’.”

The patient’s possible response to initial treatment was provided (the scenarios), ranging from total and quick improvement to deterioration. The six clinical management alternatives in relation to the continued clinical strategy, from which the respondents could choose, included choices such as brief continued care, maintenance care, the seeking of additional assistance, and complete discharge from care. To answer the questions in this questionnaire the respondents needed to take more factors into account than with the neck pain questionnaire. We selected three scenarios (1, 4, 8 and 9) for this study.

Scenario 1 describes the attack of LBP as being of 2 days duration with no previous history of LBP with complete remission after 2 visits. The patient is uncomplicated and is able to self-manage. This case indicates a person without a background of persistent or recurrent LBP, with a quick recovery and a psychological profile that indicates a good prognosis. The ‘indicated’ choice was to discharge the patient as no further treatment or referral is required, i.e.; a “non-indication” of continued care. Students who chose to keep on treating this patient by selecting the options of ‘maintenance care’ or ‘try something else’ were thus designated as delivering ‘non-indicated’ treatment and would be best described as over-servicing.

Scenario 4 describes a patient who improves with treatment with a history of a few uncomplicated episodes of acute LBP that completely resolves. The correct choice in this case is to elect some form of ‘maintenance care’., i.e. continued treatment is “indicated”.

The patient in Scenario 8 is not really exhibiting a positive response to the treatment and is getting worse. A 12-month history of intermittent LBP and 6 consultations in 1 month with a worsening profile is not a normal pattern. Despite the fact that there are no (obvious) ‘red flags’ a referral for a second opinion because some type of underlying explanatory condition could have been missed, is the correct choice. Students who chose to keep on treating this patient by selecting the options of ‘maintenance care’ or ‘try something else’ were designated as delivering ‘contraindicated’ treatment.

In Scenario 9 the patient is not improved at all and there is no obvious (biomechanical) explanation for the intermittent pattern. There are no ‘red flags’ but there is a need to consider if there might not be an underlying depression or some other disease. A second opinion is required. Any continued treatment would be ‘non-indicated’ and would also be described as over-servicing.

### Analysis

Data were entered and analysed in SPSS v.22 (IBM Corp, Armonk NY, USA) after identifying and correcting any incomplete or corrupt data. Survey items were dummy variable coded.

Responses to the two questionnaires were shown for general information but were not further discussed in this report. These were shown in tables, in which the “correct” answers were highlighted and the most commonly selected answers indicated in bold.

For the purpose of finding answers to our specific research questions, we selected items that most clearly would require care or that could be considered non-indications or contra-indications to continued care. Percentages were calculated for each of these responses and reported by year of study.

Thereafter, all correct answers for the ‘indicated’, ‘non-indicated’ and ‘contraindicated’ scenarios were displayed in a table as percentages together with their 95% confidence intervals (CIs).

Confidence intervals provide information about a range in which the true value lies with a certain degree of probability, as well as about the direction and strength of the demonstrated effect. This enables conclusions to be drawn about the statistical plausibility and clinical relevance of the study findings [24, 25].

We expected that Year 5 would have higher estimates than Year 3 and perhaps Year 4. Thus differences in estimates between study years and types of indications were identified and these were considered to be statistically significant if their 95% CIs did not overlap.

## Results

### Descriptive information

Of a possible 142 3rd, 4th and 5th year chiropractic students from Program A, 90 (63%) completed the survey and from Program B 124/ 518, giving a total of 214 students (41%), of which 54% were male (114) (Table 1). Because some students studied across multiple years and the manner in which these data were recorded at Program A, a few participants could not be placed within a specific year.

**Table 1:** Institution, year of chiropractic program, sex and percentage of participants in a survey on Australian chiropractic students (*N* = 214)

Year of Program	Males/Females	% of respondents by year
3rd year Program A	42/20	**
Program B	19/22	62%
4th year Program A	34/25	**
Program B	6/21	79%
5th year Program A	3/0	**
Program B	12/10	55%
All Years Program A Program B	79/45 37/52	** 44%

**Data could not be provided because of the inability to ascertain students’ year of study

As reported elsewhere [18] there was no difference in the psychological variable scores between institutions or between sexes, whether tested by year of program or for the whole institutional sample. Consequently, the two programs were combined for all subsequent analyses.

### General information

The results for the whole questionnaires are shown in Table 2 for the neck pain study and in Table 3 for the LBP study. Results for the research questions are found in Tables 4, 5 and 6.

**Table 2:**
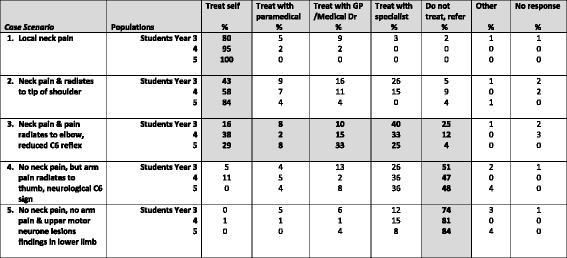
Proportion of Australian chiropractic students in Years 3,4 and 5 participating in a survey on how to choose a treatment strategy for five different neck pain scenarios (*N* = 214). For ease of comparison, results from a previous study on French chiropractors [7] have been included

The most common answer for each scenario has been written in bold. Shaded areas denote the a priori choices of the original research team as the correct or ‘indicated’ answer [19]. ‘Paramedical’ denotes an osteopath, physiotherapist nurse or another chiropractor

**Table 3:**
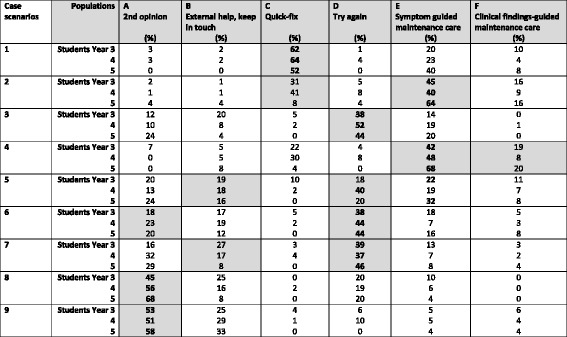
Proportion of Australian chiropractic students in Years 3, 4 and 5 participating in a survey on how to choose a treatment strategy for nine different low back pain scenarios (*N* = 214)

The most common answer for each scenario has been written in bold. Shaded denotes the a priori choices of the original research team as the correct answers for each scenario [20]. High scores that do not match the pre-selected ‘correct’ treatment strategies are also presented but are not shaded

**Table 4:** Three case scenarios for which continued care would be the correct choice in a survey of 214 Australian chiropractic students

Case Scenarios	Population	‘Correct’ response	
		N (%)	[95% CI]
Neck Scenario 1 *Simple uncomplicated neck pain*	Students Year 3 4 5	81 (80) 82 (95) 25 (100)	[71–88] [89–99] [86–100]
Neck Scenario 2 *Simple uncomplicated neck pain with pain in the trapezius*	Students Year 3 4 5	43 (43) 50 (58) 21 (84)	[33–53] [47–69] [64–96]
LBP Scenario 4 *Recurrent LBP over 12 months with previous episodes and complete recovery*	Students Year 3 4 5	58 (61) 48 (57) 22 (88)	[51–71] [46–68] [69–98]

**Table 5:** Two case scenarios for which continued care would be not be indicated (‘non-indicated’) in a survey of 214 Australian chiropractic students

Case Scenarios	Population	‘Correct’ response	
		N (%)	[95% CI]
LBP Scenario 1 *Complete recovery with no previous episode*	Students Year 3 Year 4 Year 5	62 (63) 55 (64) 13 (52)	[53–73] [53–74] [31–72]
LBP Scenario 9 *Absence of improvement for no apparent reason, probably concomitant depression*	Students Year 3 Year 4 Year 5	51 (53) 42 (51) 14 (58)	[43–63] [40–62] [35–76]

**Table 6:** Two case scenarios for which continued care would be contraindicated in a survey of 214 Australian chiropractic students

Case scenarios	Population	‘Correct’ response	
		N (%)	[95% CI]
Neck Scenario 5 *Neck pain & arm pain gone but Upper Motor Neuron lesion findings present in the lower limbs*	Students Year 3 4 5	75 (74) 70 (81) 21 (84)	[65–82] [72–89] [70–98]
LBP Scenario 8 *Absence of improvement and worsening pain*	Students Year 3 Year 4 Year 5	43 (45) 47 (56) 17 (68)	[35–55] [45–68] [47–85]

### Overall impression

The percentages of ‘correct’ choices varied between the questions (43 to 100%). There were 7 scenarios reported for students in years 3, 4 and 5. This resulted in 21 scores of which 13 were correct in less than 70% of respondents and 8 in 70% or above of students.

Objective 1

- Is there a progression in chiropractic students’ ability in different years of the program to correctly identify continuation of ‘indicated’ care?

For the three scenarios relating to indications for continuing care, the percentage scores of ‘correct’ choices for years 3, 4 and 5 ranged from 43% to 100%. Thus for nine scores, five were below 80% and four were 80% or above.

The ‘correct’ estimates increased for year of study in all three scenarios (Table 4). This was statistically significant as the 95% Confidence Intervals did not overlap when comparing the 3 years of study in these scenarios. This was not in a stepwise manner.

Thus, students were comfortable to continue to treat a simple case of uncomplicated neck pain. Likewise, they would continue treating a simple case of neck pain that spreads to the trapezius muscle. Finally, students knew to continue to treat a patient who completely recovered from an episode of LBP with a history of previous episodes.

(b) Is there a progression in chiropractic students’ ability in different years of the program to correctly identify the need for the cessation of ‘non-indicated’ care?

The percentage scores for the two scenarios relating to stopping ‘non-indicated’ care ranged from 51 and 64%. Two were above and four were below 60%.

The ‘correct’ estimates did not increase for year of study in these two scenarios (Table 5). Specifically, students did not improve in selecting the ‘correct’ choice of stopping ‘non-indicated’ care in a patient without a previous history of LBP, which had recovered completely within two visits from an acute episode. Nor did they do well in the LBP case where there was an absence of improvement for no apparent reason, but probably due to or concomitant to depression.

(c) Is there a progression in chiropractic students’ ability in different years of the program to correctly identify the need for the cessation of ‘contraindicated’ care?

For the two scenarios that related to ‘contraindicated’ care, ‘correct’ percentages ranged from 45 to 84%. The scores for the LBP scenario were all below 70% while all the neck pain scores were 70% or above.

The ‘contraindicated’ estimates increased with year of study in both scenarios but not significantly. This means that it is possible that students improve in their ability to correctly stop ‘contraindicated’ treatment but that this did not show for a patient who deteriorates with neck pain to develop absence of neck pain or arm pain but has upper motor neuron lesion findings in the lower limbs. Likewise for the LBP case, where the patient does not improve but worsens.

Despite these obvious contraindications to continue care, between 23% (Year 3) and 12% (Year 5) were still prepared to keep on treating the neck and as many as 55% (Year 3) and 32% (Year 5) would continue with the LBP case.

Objective 2. Do students understand equally well when treatment is ‘indicated’, ‘non-indicated’ and ‘contraindicated’.

- In year 3
- In Year 4
- In Year 5

### Overall impressions

As can be seen in Tables 4, 5 and 6, for all the 3 years, by far the best responses were for the simple neck pain scenario, which the vast majority considered a good indication for continued care. The second best scenario was the most severe neck profile with findings of an upper motor neuron lesion. Findings in addition to this are reported below.

2 (a) 3rd year chiropractic students;

Students were by far best at detecting an indication for continued care in the simple uncomplicated neck pain scenario (80%). However, the addition of pain in the trapezius was spotted by less than half as an indication (43%). That absence of LBP improvement with probable depression is a non-indication for continued care was understood by 53% and that absence of improvement with worsening of pain was actually a contraindication was understood by only 45%.

2 (b) 4th year chiropractic students;

Again the LBP non-indication and contraindication were not recognised by almost half, 51% and 56% correct answers, respectively. In addition, only 64% acknowledged that the person with LBP, complete recovery and no previous episodes was not an indication for continued care.

2 (c) 5th year chiropractic students;

The fifth year students, however, were very good at knowing when to continue care (88 to 100%), reasonable at understanding that LBP that fails to improve and gets worse is a contraindication to care (68% correct answers) but often unable to identify non-indications (52 and 58% correct answers).

### Post hoc analyses

Post hoc analyses of all variables per year of study were done, cross-tabulating all variables in two by two tables, which in general revealed no associations between individuals who gave the incorrect answer in one scenario and an incorrect answer in the others (data not shown).

## Discussion

### Summary of findings

This appears to be the first study to investigate clinical reasoning in chiropractic students. For this we used two clinical cases, one on LBP and one on neck pain, with different scenarios that indicated that patients should either receive continued care, were unsuitable for continued care because it would be ineffective, or that care was likely to worsen the condition or markedly prevent other necessary treatment, hence contraindicated.

We found that students were good at identifying indications to continue care and that the results, generally, got better with year of study. However, the scenarios that reflected non-indication for continued care had much worse results and did not improve in higher years. Encouragingly, for an obvious contraindicated neck scenario, the results were good from the beginning and got better but for a contraindicated LBP scenario the results started rather badly in year 3 then improved over the program years.

Incorrect clinical choices did not cluster around the same students to a significant extent.

### Explanation of findings

The best results were attained for extreme cases, especially for the neck scenario with pathological sign posts. The LBP cases required decisions without any such ‘hard’ clinical evidence. Rather, they were based on more equivocal symptoms such as the number of past episodes and fluctuating levels of improvement. It is therefore possible that the students, who had little or no clinical experience, would struggle to find the ‘correct’ answers.

The students got better at choosing to continue care but they also considered continued care when this was not indicated. Research with medical students has suggested they become over confident with training [26]. This may be an explanation for this finding in chiropractic students. In fact, students from all years were not good at stopping ‘non-indicated’ care. It therefore seems that the educative process has been unable to prepare approximately half of the students, who were probably of the attitude “try it and see how it goes” for ‘non-indicated care’. Another explanation is that students were not taught when to stop. Some may not regard this as a safety issue or being acutely dangerous but unwarranted treatment has financial implications for the patient and society and runs the risk of entrenching pain behaviours and practitioner dependence if continued for a protracted period. In other words, this invites over-servicing. If this is the case, chiropractic educators may need to face the challenge of training chiropractic students about the more subtle aspects of patient care as well as developing a healthy self-doubt.

As stated in the introduction, CCE accreditation standards have little regulatory expectations of chiropractic programs using the specific terms contraindicated or non-indicated care. A positive step would be the adoption and enforcement of these terms into the standards of CCEs and their inspection processes. Perhaps this could address the genesis of deficient chiropractic practices and profiles, such as a minority of students believing that disease is caused by ‘vertebral subluxation complexes’ and that chiropractic spinal adjustments are an effective primary treatment for diseases including AIDS and cancer [27].

Some chiropractors use so called maintenance care as secondary or tertiary prevention. However there are wide variations in its use [28]. Some have few maintenance care patients while others have many [29]. No doubt, based on common sense there seems to be a consensus for when ongoing care is indicated. This was confirmed in a survey which showed that maintenance care is thought to be useful for patients who improve well with treatment and have a history of repeat episodes [30]. On the other hand, for patients with no past episodes, maintenance care is not indicated, nor is it indicated when there is no improvement [28].

From an educational perspective, it is our observation that clinical chiropractic education centres on the initial screening of patients to avoid contraindications to care. Once this step is achieved then there is emphasis on the technical diagnosis, i.e. where the problem is and how to treat. But perhaps we do not place adequate emphasis on the different clinical trajectories observed or explaining and discussing what to expect and how to match our treatment approach including the cessation of care. This is important as LBP, for example, has several types of trajectories [31–33] and each should be considered from a common sense point of view, as, generally, there is no evidence on what sort of approach is best for which type of trajectory.

### Concerns

Despite these encouraging results, it is of concern that in our student sample approximately 20% would not stop treatment when there were serious neurological signs present [19]. This suggests that there is scope for future investigations exploring how educators could best construct educational interventions, which address these types of clinical scenarios.

We contend that common sense is a valuable clinical asset and that the discontinuation of care for a non-responsive patient clearly falls into this domain. The fostering of such an approach may go a long way to addressing the perception of over servicing.

None of the five Councils on Chiropractic Education used the term ‘non-indication’ in their accreditation standards. This study suggests that incorporation of the words ‘indication’, ‘contraindication’ and ‘non-indication’ is warranted at this level and may offer an assessable metric for use as an outcome measure for program evaluation and monitoring student progression. Consequently, it has the potential to produce more responsible graduates and enhance the public perception of chiropractic care.

## Methodological considerations

This study was cross-sectional in nature. It is therefore possible, but not likely, that the results in the different years can be explained by a cohort effect. Further studies would reveal if this is the case.

The two sets of questions (neck and LBP questionnaires) were tested and refined prior to being used in previous studies [19, 20], which supports their user-friendliness and clinical relevance. However, the two questionnaires used different formats, and it is possible that the LBP questionnaire was more difficult for students, who do not have enough clinical experience to be able to deal with several issues or aspects at the same time.

The response rate was relatively good for one chiropractic program but not so for the other. Since the study was anonymous, we could not compare responders to non-responders. However, the profiles on other factors were similar in the two programs [18]. We therefore assume that the two student samples were not biased in any particular direction. Whether they were entirely representative of their study populations is not known.

Overall, there was a small number of 5th year students. This created wide confidence intervals that may have impacted on our findings. A larger study using Australian chiropractors would clarify this and the impact clinical experience may have on some of the scenarios for the neck and LBP case.

## Conclusion

Students generally made appropriate clinical choices for when to treat. This was also the case for contraindications especially when there are obvious pathological findings. These skills were more apparent in the higher years of study. However, the concept of non-indication may not have been as well understood and did not differ between the years. This is surprising, as non-indications are essentially common-sense decisions.

## Recommendations

CCEs should adopt the terms contraindication, indication and non-indication in their accreditation standards to improve decision making on whether or not to continue care. This study suggests that there are ways to measure these indicators and that it could be used as evidence of undergraduate and graduate competency.

If student milestones require such knowledge, then more emphasis in education should be put on the indications for long-term management, especially in relation to past history and treatment outcome to avoid delivering unnecessary care.

There was a lack of improvement over the program duration for ‘non-indicated’ care. One way for chiropractic educators to improve this may be to teach the students to take a common sense approach to help students understand this concept better, including their use as a valuable clinical asset.

## Additional files

Additional file 1: Anonymous Questionnaire for Chiropractic Students Survey. (DOCX 533 kb)

## Abbreviations

GP: General or Medical Practitioner
LBP: Low back pain
UMN: Upper Motor Neuron
