# Supplementary material for: Chiropractic student choices in relation to indications, non-indications and contra-indications of continued care
Source: Chiropr Man Therap. 2018 Jan 23;26:3. doi: 10.1186/s12998-017-0170-y (PMC5778768; doi:10.1186/s12998-017-0170-y)
Supplement: Supplementary file 1 — Anonymous Questionnaire for Chiropractic Students Survey. (DOCX 533 kb) [file 12998_2017_170_MOESM1_ESM.docx]

**APPENDIX ONE.**

**Anonymous Questionnaire for Chiropractic Students Survey**

**Participant consent**

I have read the Information letter and I agree to participate in this survey. Your answers are anonymous.

The survey should take about 10 – 15 minutes to complete.

Thank you very much for your assistance.

Stanley Innes

Lecturer Chiropractic / Health Professions

Murdoch University

**Please answer the following questions**

Sex: Male  Female

Year of Program: Yr 1  Yr 2  Yr 3  Yr 4  Yr 5

**Continue over the page**

1. **CASE SCENARIO A:** The following question is seeking your response to a specific case history. Please read the case history and circle what you think is the most appropriate option from A through to F in each cases which are numbered from 1 to 5.

A 28-year old man, tennis player by profession, consults you for a right-sided intense neck pain without any radiating pain. You note an antalgic position of the head, no other musculoskeletal signs (no torticollis), no other health problems in particular, normal x-rays for his age, and there are no red flags.

In each of the cases described below, what would you do?

**Answer Options:**

1. I would treat the patient on my own.
2. I would treat the patient with the assistance of some paramedics & / or physiotherapist.
3. I would treat the patient with the assistance of a general practitioner.
4. I would treat the patient whilst asking the opinion of a specialist such as a neurologist / rheumatologist.
5. I would not treat the patient but refer him out.
6. Other, please explain at the bottom of the page.

1. “Physical examination: very tense cervical musculature, no neuro-vascular problems, right C5-6 painful on palpation, pain 7/10 on a visual analogue scale”. **Answer A B C D E F other ____________________**


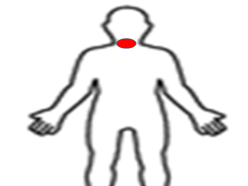


2. “Five days later the patient comes back to you: Same clinical signs but the pain now radiates into the right shoulder”. **Answer A B C D E F other ______________________**


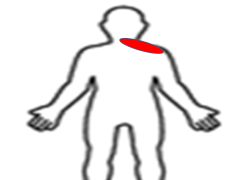


3. “Four days later: An MRI reveals a postero-lateral disc herniation at C5-6 which affects the C6 nerve root. There is now a neurological sign: C6 reflex diminished (1+), normal myotomes and dermatomes.”

**Answer A B C D E F other___________________________**


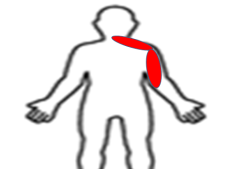


4. “Another 4 days later: The neck pain is gone. The neurological signs are obvious: C6 reflex absent (graded as 0), the C6 myotome diminished (graded as 2), C6 dermatome disturbed (reduced sensitivity).”

**Answer A B C D E F other ___________________________**


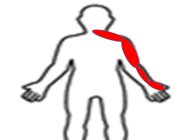


5. The pain drawing is the same and the accompanying text said: “Ten days later: The symptoms and signs are the same as last time but in addition the following is noted: the right leg shows hyperreflexia (graded as 3+), a positive

sign of Babinski on the right and slight hypoaesthesia of the right leg.”

**Answer A B C D E F other____________________________**


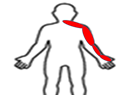


1. **CASE SCENARIO B**

*Please read the following cases and for each case, give the answer that you consider fits best with the decision you would make in a clinical setting.*

*We have selected an imaginary patient, as described in the box below. Then, different scenarios for this patient are outlined, and you are asked to select ONE of several clinical solutions (A,B,C, etc) as listed in bold letters below.*

*You can choose between the following possibilities for each of the cases presented below:*

1. **I would refer the patient to another health care practitioner for a second opinion.**
2. **I would advise the patient to seek additional treatment whilst following the patient.**
3. **I would tell the patient that the treatment is completed but that he is welcome to make a new appointment if the problem returns.**
4. **I would not consider the treatment to be fully completed and would try a few more treatments and perhaps change my treatment strategy, until I am sure that I cannot do any more.**
5. **I would follow this patient for a while, attempting to prolong the time period between visits until either the patient is asymptomatic or until we have found a suitable time lapse between checkups to keep the patient symptom-free.**
6. **I would recommend that the patient continues with regular visits, as long as clinical findings indicate treatment (eg spinal dysfunction/subluxation) even if the patient is symptom-free.**
7. **None of the above. (Please explain at the bottom of this page in legible handwriting).**

These are the basic facts for our hypothetical patient.

A 40 year old man who consults with you for low back pain with no additional spinal or musculoskeletal problems, and with no other health problems.

His x-rays are normal for his age. There are no “red flags”.

The case above could proceed in the **following nine ways** described on the next page.

**
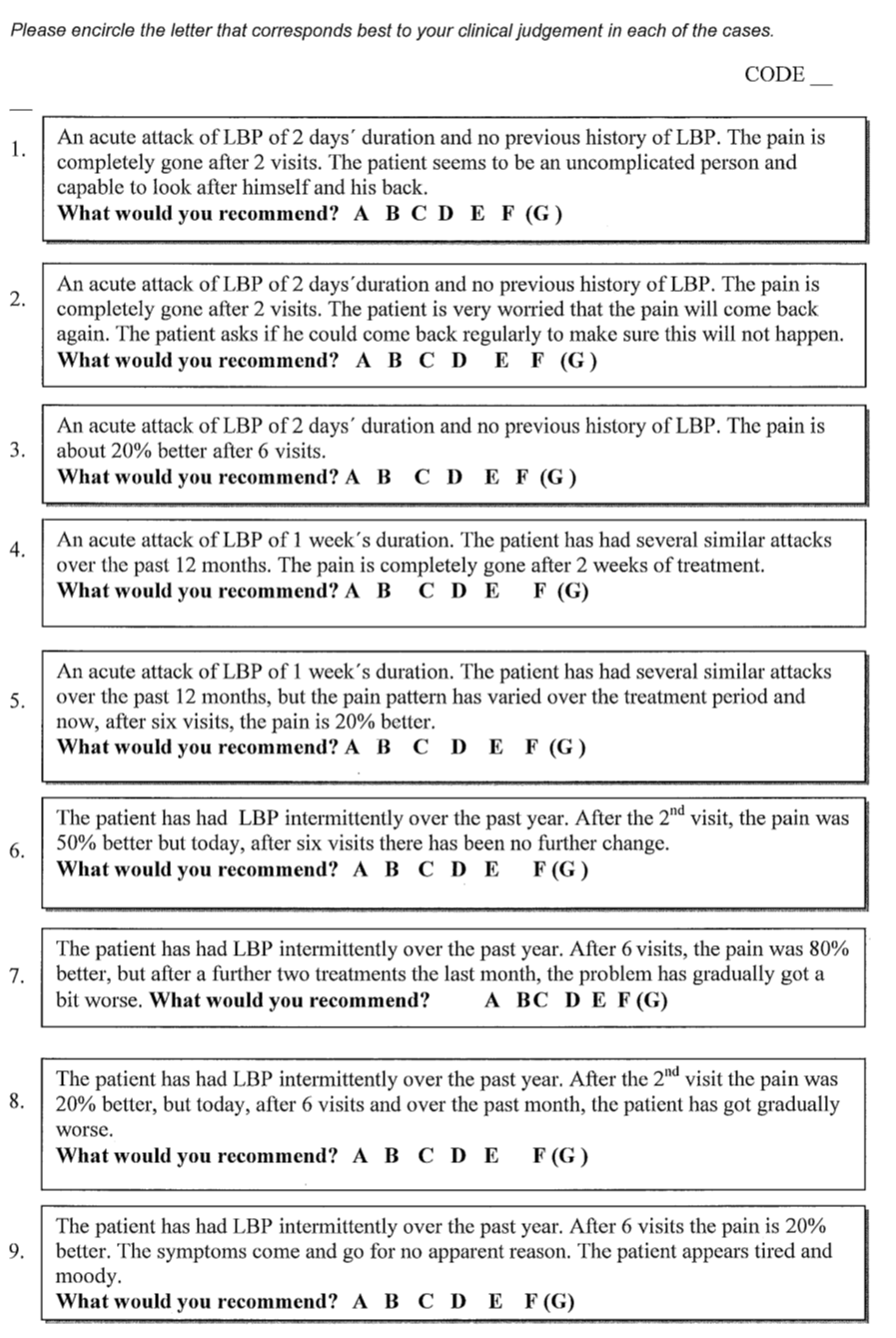
**

**Additional File 1.**

**Neck Pain Case** [7].

Below is a description of the 5 specific management strategies for the neck pain case scenario receiving chiropractic care from which the participants in the survey could select one for each of 5 scenarios. The history was for a 28-year old man, tennis player by profession, presenting with right-sided intense neck pain.

The five options were :

1. I would treat the patient on my own.
2. I would treat the patient with the assistance of some paramedics & / or physiotherapist.
3. I would treat the patient with the assistance of a general practitioner.
4. I would treat the patient whilst asking the opinion of a specialist such as a neurologist / rheumatologist.
5. I would not treat the patient but refer him out.
6. Other, please explain at the bottom of the page.

A description of the 5 scenarios, together with the clinical reasoning (cases 1-5) of the research team for the preferred management strategy for each scenario is provided below.

Case 1. “*Physical examination: very tense cervical musculature, no neuro-vascular problems, right C5-6 painful on palpation, pain 7/10 on a visual analogue scale*”.

According to the research team, this case indicates a person without a background of persistent or recurrent neck pain. It is uncomplicated and with no poor psychological profile which is indicative of a good prognosis. The team would have selected strategy A (‘I would treat the patient on my own’). As such referral or co-management was unwarranted and deemed to be an unnecessary strategy and an incorrect response.

Case 2. “*Five days later the patient comes back to you: Same clinical signs but the pain now radiates into the right shoulder*”.

Although the pain had spread to the shoulder, there was no other additional neurological progression or other signs or symptoms of deterioration. This case was thought by the team as best being thought of as uncomplicated non-specific neck pain. As such referral or co-management was unwarranted and deemed to be an unnecessary strategy. Thus the strategy the team would have selected would have been A (‘I would treat the patient on my own’).

Case 3. “*Four days later: An MRI reveals a postero-lateral disc herniation at C5-6 which affects the C6 nerve root. There is now a neurological sign: C6 reflex diminished (1+), normal myotomes and dermatome*s.”

The thoughts of the research team were that this patient had not improved as should be expected. Conservative care had not generated any positive response and hard neurological signs as well progressively deteriorating symptoms were now evident. The team felt that a change of strategy, referral or second opinion would be a reasonable clinical decision. Thus they would have selected B, C, D, or E. However, the DTR C6 reflex could be potentially regarded as equivocal, thus the response of A was thought not to be unreasonable.

Case 4. “*Another 4 days later: The neck pain is gone. The neurological signs are obvious: C6 reflex absent (graded as 0), the C6 myotome diminished (graded as 2), C6 dermatome disturbed (reduced sensitivity)*.”

This patient is not improving at a level that should be expected despite the number of visits. The patient is resistant to the type of treatment that has been provided so far. There has been evidence of progressive neurological deterioration and symptomatology. The team determined that more treatment was not warranted. Further, the patient should be referred out and that this referral should have the capacity for advanced imaging as well as more aggressive interventions. Thus the team determined the strategy should have been E (‘I would not treat the patient but refer him out”). Any other choice was deemed to be incorrect.

Case 5. *The pain drawing is the same and the accompanying text said: “Ten days later: The symptoms and signs are the same as last time but in addition the following is noted: the right leg shows hyperreflexia (graded as 3+), a positive sign of Babinski on the right and slight hypoaesthesia of the right leg*.”

There has now been a considerable time lapse with significant and serious signs and symptoms. The team determined that this was now a serious scenario well beyond the scope of conservative care and would require immediate referral. The team would have selected E as the appropriate strategy (‘I would not treat the patient but refer him out”). Any other choice was deemed to be incorrect.

**Low Back Pain Case** [8].

A description of nine scenarios (cases 1 – 9), together with the clinical reasoning of the research team, and a description of their preferred management strategy for each scenario (not included in the questionnaire) is provided below.

**Inappropriate “Referral” Strategy; Scenarios 1, 2, and 4.**

*Case 1. An acute attack of LBP of 2 days’ duration and no previous history of LBP. The pain is completely gone after 2 visits. The patient seems to be an uncomplicated person and capable to look after himself and his back.*

According to the Axen et al., 2008 research team, this case indicates a person without a background of persistent or recurrent LBP, with a quick recovery and a psychological profile that indicates a good prognosis. The Axen et al., team would have selected strategy C (“I would tell the patient that the treatment is completed but that he is welcome to make a new appointment if the problem returns”).

*Case 2. An acute attack of LBP of 2 days’ duration and no previous history of LBP. The pain is completely gone after 2 visits. The patient is very worried that the pain will come back again. The patient asks if he could come back regularly to make sure this will not happen.*

The thoughts of the Axen et al., research team were that, ideally, this patient should be dismissed, similarly to the case above (strategy C). However, the psychological profile of this patient needs to be taken into account and he should be provided with a sense of security whilst guided by the chiropractor and gradually weaned off to prevent dependency upon chiropractic treatment. The Axen et al., team therefore selected strategy E, with the intent of using a couple of more visits to improve the patient’s self-confidence.

*Case 3. An acute attack of LBP of 2 days’ duration and no previous history of LBP. The pain is about 20% better after 6 visits.*

This patient was not improving at a level and rate that should be expected. Because the basic case states that there are no red flags, the Axen et al., team decided that this case should be reconsidered and a few more attempts made. The strategy that best suited for this scenario was D.

*Case 4. An acute attack of LBP of 1 week’s duration. The patient has had several similar attacks over the past 12 months. The pain is completely gone after 2 weeks of treatment.*

This is a recurrent problem according to the past history. If the patient considers that the chiropractic treatment shortened the duration of the typical attack, he should simply return as soon as a new problem is felt to commence. Unfortunately, many patients will fail to do so, thinking that the treatment did not help when it starts up again. The Axen et al., team decided that it might therefore be advantageous to keep an eye on the patient for a while with the intent of finding out if each event of LBP can be quickly and efficiently treated at a “cost-effect “ time interval (strategy E) or if it is possible to prevent further events (strategy F).

**Inappropriate “Management” Strategy; Scenarios 6, 7, 8 and 9.**

*Case 5. An acute attack of LBP of 1 week’s duration. The patient has had several similar attacks over the past 12 months, but the pain pattern has varied over the treatment period and now, after six visits, the pain is 20% better.*

This patient is not improving at a level that should be expected despite the large number of visits, indicating that he may be resistant to the type of treatment that has been provided so far. Axen et al., decided that a change of strategy would be required (strategy D) or if the patient is referred out, it would be relevant to keep in touch to be able to be of support in the continued process (strategy B).

*Case 6. The patient has had LBP intermittently over the past year. After the 2^nd^ visit, the pain was 50% better but today, after six visits there has been no further change.*

The study by Axen et al., interpreted this patient as having reached his optimal stage with the present type of treatment and the therapy should, at this stage, either be reconsidered “in-house” or by someone else, indicating strategy A or D.

*Case 7. The patient has had LBP intermittently over the past year. After 6 visits, the pain was 80% better, but after a further two treatments the last month, the problem has gradually got a bit worse.*

The Axen et al., team used the following reasoning: The improvement seen, to date, may have been independent of the treatment and merely an expression of the typical intermittent pain pattern, or the treatment did have an effect but there is something that re-aggravated the condition. The Axen et al., team therefore reconsidered the case (strategy D) or sent the patient out for an adjunctive approach, such as training, whilst keeping in touch (strategy B).

*Case 8. The patient has had LBP intermittently over the past year. After the 2^nd^ visit the pain was 20% better, but today, after 6 visits and over the past month, the patient has gradually got worse.*

This patient has not really exhibited a positive response to the treatment and is, in fact, getting worse. That the patient is gradually worsening is not a normal pattern. Despite the fact that there are no (obvious) red flags the team would refer the patient for a second opinion (strategy A), because some underlying explanatory condition could have been missed.

*Case 9. The patient has had LBP intermittently over the past year. After 6 visits the pain is 20% better. The symptoms come and go for no apparent reason. The patient appears tired and moody.*

This patient has not improved at all and there is no obvious (biomechanical) explanation for the intermittent pattern. There are no red flags but there is a need to consider if there might not be an underlying depression or some other disease, after all. The team would not hesitate to refer out for a second opinion (strategy A).
